# Endoscopy-assisted transoral resection of the styloid process in Eagle's syndrome. Case report

**DOI:** 10.1186/1746-160X-8-21

**Published:** 2012-07-30

**Authors:** Fumihiko Matsumoto, Kaori Kase, Misato Kasai, Hiroaki Komatsu, Takako Okizaki, Katsuhisa Ikeda

**Affiliations:** 1 Department of Otorhinolaryngology, Juntendo University Faculty of Medicine, 2-1-1 Hongo, Bunkyo-ku, Tokyo 113-8421, Japan

**Keywords:** Eagle's syndrome, Three dimensional computer tomography, Transoral approach, Endoscopy

## Abstract

Eagle's syndrome is often associated with elongated styloid process or ossified stylohyoid or stylomandibular ligament. Patients with this syndrome present with recurrent cervicofacial pain. Surgical removal of the elongated styloid process is a standard treatment and can be accomplished through either a transoral or extraoral approach. Both approaches have advantages and disadvantages, and the best surgical approach remains controversial. In our case, the elongated styloid process was removed by transoral approach assisted by endoscopy. Endoscopy provides clear surgical view thus lessen the chance of neurovascular injury and other intraoperative complications. Endoscopy-assisted transoral resection is an optional alternative surgical procedure for Eagle's syndrome.

## Background

The clinical features of Eagle's syndrome were first described by Eagle in 1937 [1]. The syndrome is characterized by recurrent throat pain, pharyngeal foreign body sensation, dysphagia, referred otalgia, and neck pain. The cause of these symptoms is considered to be elongated styloid process, or ossified stylohyoid or stylomandibular ligament.

Since the diagnosis is not easy, the incidence of Eagle's syndrome in the general population may be underestimated [2]. Since the symptoms vary and are non-specific, patients with the Eagle's syndrome seek treatment in different clinics such as otolaryngology, family practice, neurology, neurosurgery, psychiatry, and dentistry. Three-dimensional computed tomography (3D-CT) has several advantages over conventional coronal and axial CT images, including providing accurate anatomical images and the relation between adjacent tissues, and is thus potentially useful for the diagnosis of Eagles syndrome. A number of groups have advocated the use of 3D-CT for radiologic examination of patients with Eagle's syndrome.

Eagle's syndrome can be treated pharmacologically or surgically, or both. Surgical treatment involves decompression of the glossopharyngeal nerve by resection of the elongated styloid process, which may be accomplished through either a transoral or extraoral approach [3-5]. Each of these two approaches has advantages and disadvantages, and the best surgical approach for Eagle's syndrome remains controversial. We report here a case of Eagle's syndrome in whom the elongated styloid process was removed endoscopically through a transoral approach. Endoscopy-assisted transoral resection may resolve the disadvantage of the transoral approach.

## Case report

**Figure 1:**
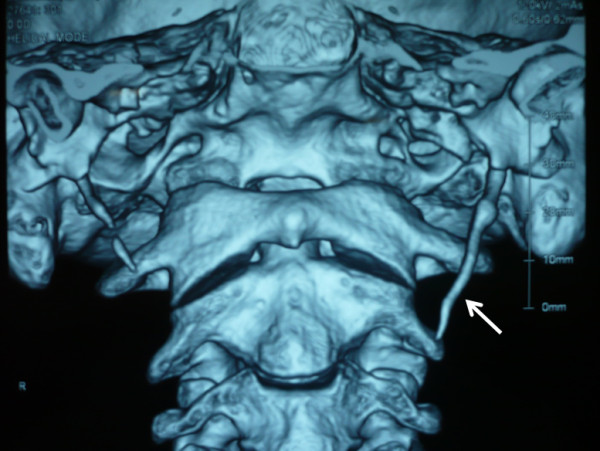
**Elongated left styloid process (arrow).** The length of left process was 44 mm, while that of the right was 25 mm.

A 42-year-old man presented with a 3-months history of right neck pain that worsened on turning to the left. He did not complain of any other symptoms such as globus sensation or odynophagia. Physical examination was negative. Palpation of the tonsils did not worsen the pain. 3D-CT reconstructed images showed a longer left process than the right. Quantitative measurements showed the left styloid process was abnormally long (44 mm), while the right was normal (25 mm, Figure 1). In view of the neck pain, the patient was diagnosed with Eagle's syndrome. The condition was explained to the patient and operative treatment was recommended. Surgical correction was made via the transoral approach. On the first postoperative visit, rotation of the head did not elicit any pain.

### *Surgical procedure* (Figure 2)

**Figure 2:**
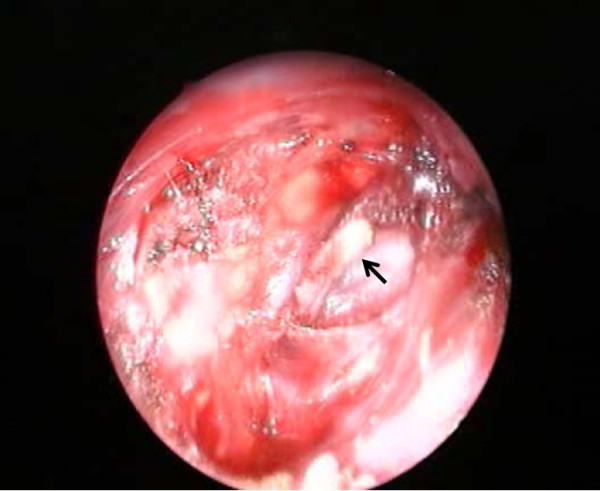
**Endoscopy provided clear vision and magnified the anatomical structures, thus allowing clear surgical field.** The styloid process was exposed and ready to remove (arrow).

The operation was performed under general anesthesia while the patient was in supine position with neck extension. An endoscope was inserted through the mouth, and the magnified surgical field allowed identification of the small vessels and nerves and allowed the surgeon to avoid injuries. Unilateral tonsillectomy was performed first, the tonsillar bed was palpated and the tip of the styloid process was identified. The mucosa was separated longitudinally at the point of palpation of the process in the tonsillar fossa. The tissue of the parapharyngeal space was carefully separated by q-tips to avoid vascular injury. Palpation was occasionally used during surgery to confirm the location of the styloid process. After exposure of the styloid process, the periosteum was incised at the tip of the process, then stripped from the tip to the base. The styloid process was excised with a bone nibbling rongeur. The styloid process must be penetrated before its removal. If the styloid process fractures accidentally before it is completely dissected, the fractured piece(s) can be pulled down through the tonsillar bed after diverging the muscles attached to it, a step that might reveal the broken piece(s). After resection of the process, the tonsillar bed was sutured with absorbable sutures to protect the parapharyngeal space from sputum at the pharynx. Oral feeding was possible the next day. Intravenous cefalotin (1 g) was injected just before the start of surgery. Follow-up examination showed uneventful recovery and no complications.

## Discussion

The actual incidence of abnormally long styloid process is probably higher than the reported incidence of 4% [6-8]. This abnormality is an important pathology in the differential diagnosis of recent increase in abnormal laryngopharyngeal sensation. The diagnostic difficulty of this condition is due to the infrequent and vague symptoms. The differential diagnosis of Eagle's syndrome includes any condition that may result in cervicofacial pain. Misdiagnosis of the syndrome can result in various unnecessary treatments. Eagle's syndrome should be suspected in the presence of persistent throat pain triggered or exacerbated by head rotation, lingual movement, swallowing, or chewing. Furthermore, exacerbation of pain during palpation of the lateral tonsillar fossa is common in this syndrome. The diagnosis is best made with a successful local injection of an anesthetic agent, and surgical intervention provides the best confirmation following postoperative cessation of all symptoms. 3D-CT images reformatted from the raw data obtained with a spiral scanner provide the necessary information on the styloid process, including its length, direction, and anatomical relation to adjacent tissues. Previous studies reported that 3D-CT is reliable for measurement of the actual length of the styloid process and the stylohyoid ligament [9-11], and helpful in establishing the diagnosis of Eagle's syndrome. In addition, 3D-CT helps the patient understand the abnormality and planned treatment. However, 3D-CT has certain disadvantages, including the fact that slight movement can degrade the images and a slightly higher radiation dose is required, depending on the number of sections taken.

The nonsurgical treatment of Eagle's syndrome generally involves pharmacotherapy with anticonvulsants or antidepressants, but the results are short-lived [12]. Long-lasting symptom relief requires the surgical removal of the long portion of the styloid process. Two approaches are used in surgical treatment, the transoral and extraoral approach. Several transoral and extraoral cervical approaches have been described for the surgical management of Eagle's syndrome [3,13]. Chase et al. [4] and Strauss et al. [14] made an interesting comparison between the transoral and extraoral surgical approaches. However, the best surgical approach for Eagle's syndrome has not yet been established. The main advantage of the extraoral approach is the adequate anatomic exposure of the process and its associated structures. The fine surgical view is important to avoid neurovascular injury. In addition, this sterile surgical technique reduces the risk of surgical site infection. The major disadvantage of the external approach is the postoperative cosmetic deformity due to scar formation. Other disadvantages include extensive facial dissection, longer duration of surgery, and uncomfortable paresthesia of cutaneous nerves such as the great auricular nerve [3,4,12,15-17]. The transoral approach involving resection of the styloid process is relatively easy to perform and leaves no external scar [4]. Both the operation and recovery times of this procedure are short. However, the rare disadvantages of the transoral approach are possible deep cervical infection, poor visualization of the surgical field and possible temporary edema at the operative site and submandibular and retromandibular regions. The poor visualization may increase the risk of neurovascular injury. Prasad et al. [12] treated 58 patients with Eagle's syndrome through the transoral approach without any major complications such as infection of the deep neck space or injury of the major vessels or nerves in that area. Certain techniques are available to reduce the risk of intraoperative and postoperative complications. First, endoscopy-assisted surgery can magnify the surgical view and thus enhance the identification of small vessels, nerves and other anatomically important structures. To maintain a clear surgical field, minimization of bleeding is essential. Cauterization of small vessels by bipolar cautery before damaging them is one of the techniques to ensure a clear view. Maintaining a good surgical view is important in avoiding neurovascular damage and injury of the important structures. The use of endoscopy seems to have few major disadvantages. In addition, palpation of the styloid process by finger is also important to confirm the location of the process. This technique avoids misdirection. The risk of cervical infection due to contamination is also minimized by closure of the tonsillar bed opening with absorbable sutures. It is generally accepted that the transoral approach should be used only if the distal tip of the styloid process can be palpated in the tonsillar fossa but, more importantly, only if the surgeon is familiar with the technique and handling of possible complications [3].

## Conclusion

Endoscopy-assisted transoral resection may resolve the disadvantage of this procedure, and represent a favorable alternative surgical procedure for Eagle's syndrome. Transoral approach assisted endoscopy is recommended for treatment of patients with Eagle's syndrome.

## Competing interests

The authors declare that they have no competing interests.

## Authors’ contributions

FM surgery performed and documented the case. Drafted the manuscript. KK: exams performed the analysis of the image. MK: exams performed the analysis of the image. HK: performed surgery. TO: review of literature. Drafted the manuscript. KI: conducted a review of literature. Drafted the manuscript.

## Consent

Written informed consent was obtained from the patient for publication of this report and any accompanying images.
